# Antimycobacterial and cytotoxicity activity of microcystins

**DOI:** 10.1186/s40409-015-0009-8

**Published:** 2015-03-20

**Authors:** Daniela Fernandes Ramos, Alexandre Matthiensen, Wilson Colvara, Ana Paula Souza de Votto, Gilma Santos Trindade, Pedro Eduardo Almeida da Silva, João Sarkis Yunes

**Affiliations:** Research Center in Medical Microbiology, Federal University of Rio Grande (FURG), Rio Grande, Rio Grande do Sul State Brazil; Brazilian Corporation of Agricultural Research (Embrapa), Concórdia, Santa Catarina State Brazil; School of Chemistry and Food, Federal University of Rio Grande (FURG), Rio Grande, Rio Grande do Sul State Brazil; Graduation Program in Physiological Sciences, Institute of Biological Sciences, Federal University of Rio Grande (FURG), Rio Grande, Rio Grande do Sul State Brazil; Laboratory of Cyanobacteria and Phycotoxins, Institute of Oceanography, Federal University of Rio Grande (FURG), Rio Grande, Rio Grande do Sul State Brazil

**Keywords:** Mycobacteria, Antimycobacterial agents, Cytotoxic activity, Microcystins

## Abstract

**Background:**

The present work aimed to evaluate the antimycobacterial activity and cytotoxicity of *Microcystis aeruginosa* toxins, the MC-LR variant and purified extract of [D-Leu^1^] microcystin-LR.

**Methods:**

The antimicrobial activity of *M. aeruginosa* extract and microcystin was evaluated by resazurin microtiter assay against *Mycobacterium tuberculosis, M. terrae, M. chelonae* and *M. kansasii*. The cytotoxicity assay was performed by trypan blue exclusion against the HTC cell line.

**Results:**

Antimicrobial activity was observed in the hexanic extract of *M. aeruginosa* (RST 9501 strain) against *M. tuberculosis*, including sensitive and resistant strains with minimal inhibitory concentrations (MIC) between 1.93 μM and 0.06 μM. The high activity of *M. aeruginosa* hexanic extract could be attributed to the major presence of the toxins MC-LR and [D-Leu^1^] MC-LR that showed activity at MIC between 53 and 0.42 μM against tested mycobacterial strains. Even at the highest concentration tested, no toxicity of *M. aeruginosa* extracts was identified against HTC cells.

**Conclusions:**

These preliminary results suggest that [D-Leu^1^] MC-LR is a promising candidate for the development of a new antimycobacterial agent.

## Background

“Nontuberculous mycobacteria” is a general expression applied for different species of the genus *Mycobacterium* that do not belong to the *Mycobacterium tuberculosis* complex [1]. They are also recognized as causative agents of opportunistic infections in humans that affect mainly patients with preexisting pulmonary diseases – such as chronic obstructive pulmonary disease or tuberculosis (TB) – or those with impaired systemic immunity [2-4]. The latter group includes patients with HIV infection, leukemia and under immunosuppressive therapy [5,6].

For most nontuberculous mycobacterial infections, treatment is based on drugs that may differ according to the causal agent, in particular between slow- (e.g. *M. avium*, *M. kansasii*) and fast-growing species (e.g. *M. abscessus*, *M. fortuitum*) [1]. In general, drug therapy is long, costly, and often associated with toxic side effects. In addition, high rates of natural antibiotic resistance are common among nontuberculous mycobacteria, which increases the challenges for new drug discovery [1,7].

TB remains a major global health problem reaching millions of people every year and ranking as the second leading cause of death among infectious diseases worldwide [8]. The current treatment available against TB establishes a multidrug regimen that lasts a minimum of six months and does not guarantee a complete eradication of the infection [9].

Furthermore, the increased number of TB cases due to multidrug resistant and extensively drug resistant strains (MDR and XDR) and HIV co-infection have pointed out the urgent need for alternative treatment. In recent years, research on the development of new anti-TB therapies has focused on novel agents from both synthetic and natural sources [10]. For thousands of years, plant-derived drugs have been empirically used in the treatment of numerous human disorders. Many conventional drugs originate from plant sources, such as aspirin (from willow bark), digoxin (from foxglove), quinine (from cinchona bark), and morphine (from the opium poppy) [11].

Marine natural products play an important role in drug development particularly in anticancer, antibiotic and antiparasitic therapies. It is well known that macrocyclic peptides may demonstrate drug-like physicochemical and pharmacokinetic properties such as good metabolic stability, solubility, lipophilicity and bioavailability [12]. More than 800 secondary metabolites belonging to several classes of substances have been isolated and identified, which includes enzyme inhibitors; photosynthesis inhibitors; antimicrobial, antimitotic, immunosuppressive and antitumor peptides [13-15].

Microcystins, more than 65 structural variants are currently known, are cyclic heptapeptides, composed of seven amino acids, namely, five non-protein and two protein amino acids. These two protein amino acids distinguish microcystins from one another, while the other amino acids are more or less constant among the variants [16-18].

**Figure 1:**
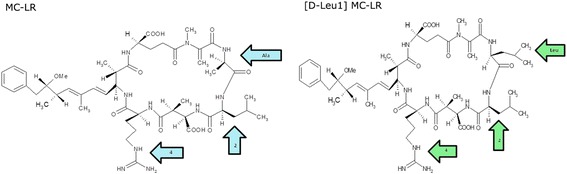
**General structure of microcystins (LR) with leucine (L) in the amino acid position 2 and arginine (R) in the amino acid position 4 and the structural differences in the position 1 of MC-LR (left) and [D-Leu** ^**1**^ **] MC-LR (right).**

Structural variations have been identified at all seven positions of the heptapeptide ring. Microcystin-LR (MC-LR) (Figure 1) is the most commonly identified cyanotoxin in environmental samples, which presents a leucine (L) and an arginine (R) respectively in X and Y positions of the cyclic heptapeptide [19,20]. The combined presence of the two L-amino acids is used in the nomenclature of the variants while the position 1, which contains D-Ala, is relatively conserved [21].

Another variant of microcystin is [D-Leu^1^] MC-LR, which contains D-Leu in position 1. This toxin was detected in cells of *Microcystis aeruginosa* (RST 9501 strain) isolated from Patos Lagoon (southern Brazil). This major waterbody has a history of extensive nuisance blooms and scums of hepatotoxic *Microcystis* [22].

The present study evaluated the antimycobacterial activity and cytotoxicity of *Microcystis aeruginosa* toxins, the variant MC-LR and purified extract of [D-Leu^1^] microcystin-LR against *Mycobacterium tuberculosis, M. chelonae, M. terrae* and *M. kansasii*.

## Methods

### *Microcystis* culture conditions

*Microcystis* RST 9501 (UPC Culture Collection, Federal University of Rio Grande) isolated from the estuary of Patos Lagoon is the [D-Leu^1^] MC-LR producing variant and was grown in BG-11 medium with nitrate as previously described [23,24].

### Preparation of *microcystis aeruginosa* extracts

The extract was prepared using lyophilized cells of *Microcystis aeruginosa* according to the protocol described by Beattie *et al.* [24]. Briefly, the cells were dissolved in absolute methanol (Sigma, USA), sonicated three times and centrifuged (10,000 × g) at 4°C, for ten minutes. Extracts were evaporated at 40°C and then redissolved in ultrapure water (Direct Q3, Millipore, USA). The other extract preparations, presented in Table 1, replaced methanol with hexane, chloroform or water. Finally, samples were centrifuged and the supernatant was collected and stored at-20°C. Microcystin content was determined using a commercial enzyme-linked immunosorbent assay (ELISA) with polyclonal antibodies (EnviroLogix Inc., USA). Different concentrations of microcystin were prepared after appropriate dilutions with phosphate buffered saline (PBS – Ca^+2^ and Mg^+2^ free). Characterization of microcystins produced by the strain RST 9501 was previously reported by Matthiensen *et al.* [17,25]. For the extraction of microcystin from cells of the strain RST 9501, the toxin [D-Leu^1^] microcystin-LR was purified from cell extracts, following Lawton *et al.* [26]. The chemical compound microcystin-LR was purchased from Sigma (USA).

**Table 1:** **Minimum inhibitory concentration (MIC) of different** ***Microcystis aeruginosa*** **extracts against** ***Mycobacterium tuberculosis*** **strains**

**Extracts**	***M. tuberculosis***					
	**H**_**37**_ **Rv**		**RMPr**		**INHr**	
	**(μM)**		**(μM)**		**(μM)**	
*M. aeruginosa* (aqueous)	R	>1.93	R	>1.93	R	>1.93
*M. aeruginosa* (hexanic)	S	≤0.06	S	≤0.60	S	0.12
*M. aeruginosa* (chloroformic)	R	>1.93	R	>1.93	R	>1.93
*M. aeruginosa* (methanolic)	S	1.93	S	0.96	S	0.96

R: resistant, S: sensitive, H_37_Rv: sensitive strain, RMPr: rifampicin-resistant strain, INHr: isoniazid-resistant strain. Extract with MIC > 1.93 μM were considered inactive.

Finally, both toxins were resuspended in water and then analyzed by high performance liquid chromatography (HPLC – Shimadzu SCL-10A_vp_, Japan) to determine the concentration of microcystins prior to tests.

### Microcystin analysis

Analysis of microcystin obtained from *Microcystis* RST 9501 was performed using the HPLC equipment Shimadzu SCL-10A_VP_ (Japan). The analysis was carried out using a C_18_ Luna (4.6 × 250 mm, 5 μm particle size; Phenomenex, USA) reversed-phase column at 40°C with UV detection at 238 nm. The mobile phase was Milli-Q water/CH_3_CN (J. T. Baker, USA), both containing 0.05 % (v/v) trifluoroacetic acid (Merck, Germany), initially at 65:35 and using a linear gradient over 20 minutes of 100 % CH_3_CN at 1 mL.min^-1^.

### Isolates and *mycobacterium* spp. preparation

The antimicrobial activity of *Microcystis aeruginosa* extract and microcystin were evaluated against *Mycobacterium tuberculosis* H_37_Rv (ATCC 27294) pan-susceptible strain and against two clinical isolate mono-resistant to isoniazid and rifampicin with *kat*G S315T and *rpo*B S531L respectively. Furthermore, *M. aeruginosa* extract, [D-Leu^1^] MC-LR, and microcystin-LR (Sigma, USA) toxins were tested against the nontuberculous mycobacteria: *M. terrae* (ATCC15755), *M. chelonae* (ATCC 946) and *M. kansasii* (ATCC12478).

The bacterial suspensions obtained of culture in Ogawa Kudoh medium for about 14 days were prepared in sterile water containing pearls of glass of 3 mm. The suspension was homogenized by vortex agitation and the turbidity was adjusted in agreement with tube one of the scale of McFarland (3.2 × 10^7^ cfu/mL). The inoculum was prepared diluting the bacterial suspension in the proportion of 1:25 in medium 7H9 broth [4.7 g of Middlebrook 7H9 broth base (BD Difco, USA) 2 mL of glycerol (Vetec, Brazil) in 900 mL of water] enriched with 10 % oleic acid-albumin-dextrose-catalase (OADC – BBL, Media Additives, USA) [27].

### Evaluation of antimycobacterial activity

The method used for the determination of the antimycobacterial activity was the resazurin microtiter assay [28]. In brief, the assay is accomplished on microplates (96 wells) using resazurin as indicator of cellular viability. Medium 7H9 enriched with 10 % OADC was employed. The extracts and pure microcystin were weighed, dissolved in DMSO and the determination of minimal inhibitory concentration (MIC) was carried out starting from 53 to 0.06 μM in serial dilutions of 1:2.

### Cytotoxicity assay

The HTC cell line was obtained from the Cell Bank of Rio de Janeiro, Brazil. HTC cells were grown in RPMI 1640 medium (Gibco, USA) supplemented with sodium bicarbonate (0.2 g/L) (Vetec, Brazil), L-glutamine (0.3 g/L) (Vetec, Brazil), Hepes (25 mM) (Acros, USA) and b-mercaptoethanol (5 × 10–5 M) (Sigma, Germany), with 10 % fetal bovine serum (Gibco, Brazil), 1 % of antibiotic and antimycotic (penicillin – 100 U/mL, streptomycin-100 mg/mL and amphotericin B - 0.25 mg/mL), in disposable plastic flasks, at 37°C.

The cytotoxicity assay was performed by trypan blue exclusion after 24 hours of incubation with microcystins. Three independent experiments were carried out using triplicates in each experiment. Data are expressed as mean + standard error and analyzed using ANOVA followed by Tukey’s multiple range test. Significance level was fixed in 0.05.

## Results

*M. aeruginosa* RST 9501 extracts were evaluated against *M. tuberculosis* pan-susceptible (H_37_Rv), rifampicin- (RIFr) and isoniazid-resistant strains (INHr). The MIC for these four extracts ranged from 1.93 μM to 0.06 μM. The aqueous and chloroformic extracts did not present antimycobacterial activity within these concentrations.

The methanolic extract had a MIC of 1.93 μM against H_37_Rv and of 0.96 μM against the tested resistant strains. The hexanic extract showed the highest activity, with a MIC of 0.12 μM against INHr, ≤ 0.60 μM against RIFr and ≤ 0.06 μM against H_37_Rv (Table 1). This high activity of the hexanic extract could be attributed to the possible greater concentration of lipophilic compounds. Therefore, the molecules of microcystins (MC-LR and [D-Leu^1^] MC-LR) were evaluated against *M. tuberculosis* H_37_Rv, RIFr and INHr strains.

The cyanotoxin MC-LR did not present any inhibitory activity on the three strains at the concentration of 53 μM. On the other hand, [D-Leu^1^] MC-LR was active with a similar MIC (13.2 μM) for susceptible and resistant *M. tuberculosis* strains (Table 2). Therefore, the isolated cyanotoxin showed a MIC up to 220 times higher than that of the hexanic extract.

**Table 2:** **Minimum inhibitory concentration (MIC) of the microcystin variant [D-Leu** ^**1**^ **] MC-LR from extracts of** ***M. aeruginosa*** **RST 9501 and MC-LR (commercially available) against** ***M. tuberculosis*** **strains**

**Microcystin variant**	***M. tuberculosis***					
		**H**_**37**_ **Rv** **(μM)**		**RMPr** **(μM)**		**INHr** **(μM)**
[D-Leu^1^] MC-LR	S	13.2	S	13.2	S	26.5
MC-LR	R	>53	R	>53	R	>53

[D-Leu^1^] MC-LR: microcystin isolated from the extract of *Microcystis* RST 9501; MC-LR: commercially obtained; R: resistant; S: sensitive; H_37_Rv: sensitive strain; INHr: isoniazid-resistant strain; RMPr: rifampicin-resistant strain. Extracts with MIC > 53 μM were considered inactive.

The two microcystins were evaluated against three nontuberculous mycobacteria showing high activity for all species tested. *M. terrae* was the most resistant to nontuberculous mycobacteria, it showed antimycobacterial activity against the two tested toxins with MIC of 6.74 μM and 1.08 μM for [D-Leu^1^] MC-LR and MC-LR, respectively (Table 3).

**Table 3:** **Minimum inhibitory concentration (MIC) of the microcystin variant from extract of the** ***M. aeruginosa*** **RS9501 and MC-LR (obtained commercially) against** ***M. tuberculosis*** **strains**

**Microcystin Variant**	**Nontuberculous**					
	**Mycobacteria**					
		***M. terrae*** **(μM)**		***M.chelonae*** **(μM)**		***M.kansasii*** **(μM)**
[D-Leu^1^] MC-LR	S	6.74	S	0.84	S	0.42
MC-LR	S	1.08	S	2.15	S	2.15

[D-Leu^1^] MC-LR: Microcystin isolated of extract of the *Microcystis* RST 9501 strain, MC-LR: obtained commercially, R: resistant, S: sensible.

A further comparison between both toxins effects suggested that microcystin MC-LR showed lower activity than [D-Leu^1^] MC-LR against *M. chelonae* and *M. kansasii,* with a minimum inhibitory concentration of 2.15 μM for the two strains, while the other variant showed minimum inhibitory concentration of 0.84 μM and 0.42 μM for *M. chelonae* and *M. kansasii*, respectively.

**Figure 2:**
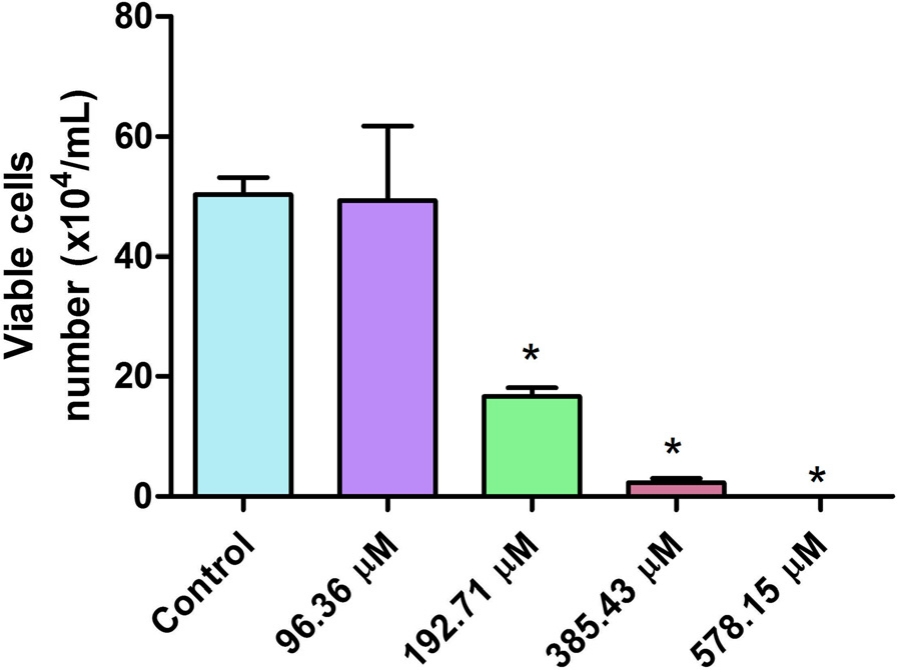
**Number of HTC viable cell by trypan blue exclusion test 24 hours after exposure to different concentrations of** ***Microcystis aeruginosa*** **strain 9501 (578.15 to 93.36** μ**M) which produces [D-Leu** ^**1**^ **] MC-LR.** Data are expressed as mean + standard error. *Indicates significant difference from the control (p < 0.05).

Exposure of HTC cells to *Microcystis aeruginosa* strain 9501 which produces [D-Leu^1^] MC-LR induces a decrease in viable cell number, as determined by trypan blue exclusion, in a concentration dependent manner, 24 hours after exposure (Figure 2). There were no differences in the number of viable cells (p > 0.05) between control and treated cells in the lowest concentration (96.36 μM). However, from the concentration of 192.71 μM on, there was significant difference (p < 0.05) in the number of viable cells, which indicates cytotoxic effect. Note that at the highest concentration (578.15 μM) no viable cell was found.

## Discussion

In recent years, there has been a growing interest in the study of natural peptide molecules, whose mode of action promises both low susceptibility to multidrug resistance mechanisms and high activity against a vast range of microorganisms [29,30]. The application of peptides for drug discovery is merited because of their ease of synthesis, large structural diversity and commonly high potency [31].

The present study identified in extracts from *M. aeruginosa* RST 9501 active compounds with antimycobacterial activity against *M. tuberculosis* growth, including sensitive and resistant strains. Several metabolites produced by cyanobacteria have been identified in the literature. There is a special interest, because these secondary metabolites are not only disease causing agents but also bioactive molecules applied to further studies [19,32-34]. Some works have identified a range of cyanobacteria compounds isolated from toxic blooms of *Microcystis*, *Anabaena* and *Nostoc* known to produce a diverse array of bioactive compounds exhibiting a broad spectrum of activity, including anticancer, antiviral, antibacterial, antifungal and anti-inflammatory activity, besides cytotoxic activities [19,32,34,35].

In this study, four *M. aeruginosa* extracts were tested, the methanolic extract was more active against resistant strains (RIFr and INHr) than sensitive *M. tuberculosis* strains. Microcystins are a group of chemically related cyclic peptides [13] and most commonly studied group of cyanotoxins,. Therefore, in order to identify the possible active compounds derived from the hexane extraction that was more active among the extracts from *M. aeruginosa* cells evaluated, the two known cyanotoxins ([D-Leu^1^] MC-LR and MC-LR) were evaluated against *M. tuberuculosis* and three nontuberculous mycobacteria species.

Between the two variants derived from the same cyanobacterial toxin, just [D-Leu^1^] MC-LR showed antimicrobial activity against three different strains of *M. tuberculosis*. However, the MIC of this variant was higher than that of the methanolic and hexane extracts. According to Pauli *et al.* [36], the differences between the extract and pure cyanotoxin activity can be attributable to the MIC of the crude extract, which may not be a reliable antimycobacterial activity indicator, since it, the activity may be to antagonist between the substances, or otherwise, synergism between them, that decreasing or increasing effects on the MIC. Moreover, an extract with high activity (lower MIC) could have several compounds with moderate antimycobacterial activity; becoming it the most active extract with a pure and isolated substance.

The activity of [D-Leu^1^] MC-LR was unchanged against strains of *M. tuberculosis* resistant to rifampicin and isoniazid compared to pan-susceptible strains. This is an important finding since the resistance to isoniazid and rifampicin comprise a major drawback of tuberculosis control programs.

Interestingly, the small antimicrobial activity of the two studied microcystins variants observed against different strains of *M. tuberculosis* was not observed for nontuberculous mycobacteria. MC-LR was less active than [D-Leu^1^] MC-LR against *M. chelonae* and *M. kansasii*. A few studies have shown that drug susceptibility of nontuberculous mycobacteria are distinct from that observed in *M. tuberculosis* because, in general, the resistance of nontuberculous mycobacteria is related to cell wall permeability and efflux pumps, specially in the presence of a specific mutation [37]. In this study, however, *M. terrae* was less susceptible against cyanotoxins. In addition, the MIC against *M. tuberculosis* strains was higher than that used against all nontuberculous mycobacteria.

The activity of these molecules against *M. chelonae* is significant, since it indicates that the low permeability limits the activity of this kind of hydrophilic molecules, which impairs treatment [38]. Regarding the mechanism of action proposed for microcystins is the inhibiting serine/threonine phosphatases 1 and 2A, which leads to protein phosphorylation and the consequence is cytoskeletal damage, liver necrosis and hemorrhage in the liver which is directly related to their cytotoxicity and tumor promoting activity [39]. However, there is only a few data on tissue concentrations of microcystins in exposed humans or animals, which were obtained after exposure to high toxic doses of microcystins [40].

In our study, a single difference in the chemical constitution of the heptapeptide was significantly important in increasing the antimycobacterial activity. In addition, considering that leucine is a hydrophobic amino acid, this characteristic may interfere in the activity of the peptide that was enhanced by presence.

According to Mandal *et al.* [41], variations of amino acid residues in peptides have received considerable attention since they alter the activity against pathogenic microorganisms, which has a significant impact on antibacterial activity [41]. A study showed a difference in their activity against *M. tuberculosis* and *Mycobacterium avium-intracellulare* by the change of only one amino acid residue in their peptide moiety [42].

Peptides may adopt secondary structures, which are responsible for their receptor affinity and biological activity. The rational design can be sufficient to endow antibacterial efficacy and to circumvent drawback effects in this potential therapeutic agent [30,31].

According to Votto *et al.* [43] and considering that microcystins may provoke oxidative stress, the difference in sensitivity of MDR and non-MDR cells can be associated with dissimilar antioxidant defenses. In this context, the higher catalase activity observed in the same work may help to explain, at least partially, the resistance of MDR cells to microcystin exposure. This MDR cell line with higher catalase activity also showed lower DNA damage than its parental cell line, suggesting the involvement of reactive oxygen species (ROS) in the toxicity exerted by this cyanotoxin. Therefore, the significant increase in ROS production observed in non-MDR cells, in contrast with MDR cells when both cell lines were exposed to microcystins, suggest that MDR cells, at least in part, were more resistant to microcystins due to a higher antioxidant competence. These authors also present other factors which may have contributed for the resistance to microcystin in MDR cells [43].

The choice of the cell line used in this study was supported by the fact that it is in the liver that microcystins are metabolized. The results show a significant sensitivity of tumoral liver cells to this substance in only 24 hours of exposure.

## Conclusions

In the present study, the antimicrobial activity of the hexanic extract from *M. aeruginosa* RST 9501 against *M. tuberculosis* – including sensitive and resistance strains, and nontuberculous mycobacteria – was observed and possibly associated with the presence of cyanotoxins. When the activity of these toxins was assessed, the variant [D-Leu^1^] MC-LR was the most active against tested mycobacterial strains. Moreover, was not identified cytotoxic activity at concentrations whose antimicrobial activity. Also, was not identified cytotoxic activity at concentrations which antimicrobial activity was observed. These results showed the importance of detailed studies on the activity of extracts and toxins derived from *M. aeruginosa* strains as promising bioactive molecules in the treatment of mycobacterial diseases.

### Ethics committee approval

The present study was approved by the Ethics Committee of the Federal University of Rio Grande.
